# *Geobacter*-based syntrophy drives biogenic element cycling in anoxic soils: a review

**DOI:** 10.3389/fmicb.2026.1932208

**Published:** 2026-09-04

**Authors:** Rong Tang, Huayuan Shangguan, Zhengli Yang, Weiguo Zhang, Yin Ye

**Affiliations:** 1 College of Ecology and Resources Engineering, Wuyi University, Wuyishan, China; 2 College of Tea and Food, Wuyi University, Wuyishan, China; 3 Engineering Research Center of Prevention and Control of Geological Disasters in the Mountainous Areas of Northern Fujian, Wuyi University, Wuyishan, China

**Keywords:** biogeochemical cycle, extracellular electron transfer, geobacter, interspecies electron transfer, microbial syntrophy

## Abstract

Microbial syntrophy is a central organizing principle in anoxic soils, where the degradation of organic matter and the cycling of nitrogen, sulfur, and iron are constrained by electron availability and thermodynamics. Among soil anaerobes, *Geobacter* species occupy a distinctive ecological anoxic niche in which they form syntrophy relationship with partner cells to exchange electrons for anaerobic carbon mineralization, methane production, nitrate reduction, sulfur and Fe(III) reduction. Here, we review how *Geobacter*-based syntrophy contributes to C/N/S/Fe cycling in anoxic soils. We first summarize the mechanisms of *Geobacter*-based syntrophy through interspecies electron transfer (IET), then detailedly evaluate how *Geobacter*-based syntrophy regulates C/N/S/Fe cycling respectively in soils with specific metabolic pathways, enzymes, and ecological outputs. We propose that *Geobacter*-based syntrophy should be viewed as a redox-partitioning mechanism that determines whether electrons from organic carbon are routed toward methane, ammonium, gaseous nitrogen, minerals, or microbial biomass.

## 1 Introduction

Soils are spatially heterogeneous redox mosaics in which oxygen, nitrate, sulfate, ferric minerals, organic matter, and reduced metabolites vary over micrometer-to-millimeter scales. Under these conditions, single microorganism could hardly complete the entire degradation and remineralization sequence of organic matters (Li et al., 2026). Instead, carbon (C), nitrogen (N), sulfur (S), and iron (Fe) cycling are easily driven by consortia in which respiratory anaerobes, fermenters, methanogens, denitrifiers, sulfate reducers, sulfur oxidizers, and metal reducers exchange electrons to form syntrophy relationships (Yu et al., 2025). Syntrophy is therefore not merely a nutritional association, but a redox cooperation strategy that allows energetically marginal reactions to proceed by coupling electron release in one organism to electron consumption in another (Kouzuma et al., 2015; Liu et al., 2018; Rotaru et al., 2014).

Among soil anaerobes, *Geobacter* species stand out as keystone organisms in anoxic soil aggregates, flooded paddy soils, wetland sediments, and subsurface environments as they are able to oxidize organic carbon and transfer the generated electrons to extracellular electron acceptors via extracellular electron transfer (EET) (Liu et al., 2024, 2023; Lovley, 2025; Ye et al., 2022). Importantly, *Geobacter* species are capable of direct interspecies electron transfer (DIET), a mode of syntrophy in which electrons are transferred directly from one microbial cell to another without diffusible mediators such as H_2_ or formate (Summers et al., 2010). DIET was first demonstrated in cocultures of *Geobacter metallireducens* and *Geobacter sulfurreducens*, where *G. metallireducens* oxidized ethanol and transferred electrons directly to *G. sulfurreducens*, which reduced fumarate. The capacity of *Geobacter* to engage in DIET with phylogenetically diverse partners, including methanogenic archaea, sulfate-reducing bacteria, denitrifiers, and Fe(III)-reducing consortia, positions this genus as a central hub connecting the biogeochemical cycles of C, N, S, and Fe in anoxic soils.

This minireview synthesizes current understanding of how *Geobacter*-based syntrophy drives elemental cycling in anoxic soils. We first summarize the molecular mechanisms by which *Geobacter* accomplish interspecies electron transfer (IET) with partner microorganisms to form electric syntrophy, then examine, element by element, the metabolic and physiological basis of *Geobacter*-based syntrophy in driving C, N, S, and Fe cycling, with emphasis on the metabolic pathways, enzymes, and regulatory strategies that underpin these processes.

## 2 The mechanisms of *Geobacter*-based syntrophy

*Geobacter*-based syntrophy could accomplished through IET, which is classified into DIET and MIET (Table S1). DIET refers to the direct exchange of electrons between microorganisms through their own conductive structures, such as electrically conductive nanowires and extracellular cytochromes c (Shrestha et al., 2013). In addition, exogenous conductive materials like magnetite, granular activated carbon, or biochar can also mediate IET between microorganisms (Huang et al., 2018). Because the IET mediated by these materials does not require the assistance of energy carriers but instead relies directly on the conductivity of the mediators for electron transfer, this type of IET is also classified as DIET. MIET, in contrast, refers to electron transfer between microorganisms via redox-active molecules produced by the bacteria themselves, such as hydrogen/formate and riboflavin, or exogenous small molecules, such as humic substances (Stams and Plugge, 2009).

### 2.1 Direct interspecies electron transfer

**Figure 1:**
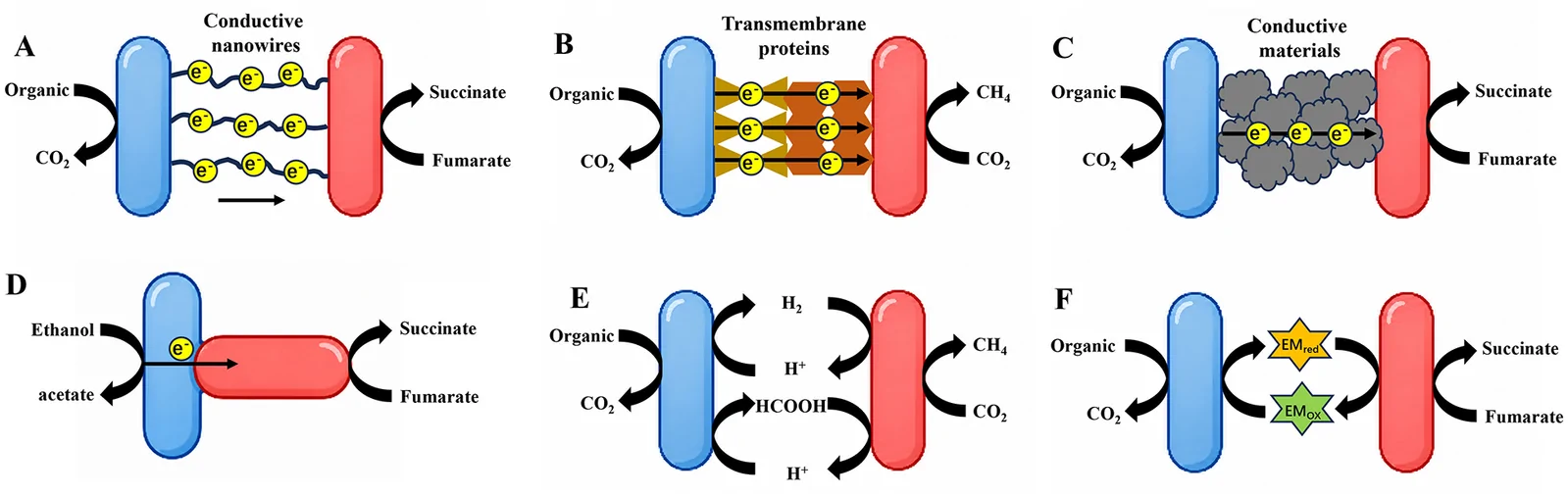
Mechanisms of IET. DIET through conductive nanowires **(A)**, transmembrane proteins **(B)**, exogenous abiotic conductive materials **(C)** and cell fusion **(D)**. MIET through hydrogen/formate **(E)** and redox-active electron mediators **(F)**.

*Geobacter* species have evolved molecular machinery for transferring electrons beyond the cell envelope through DIET (Figure 1). Study observed that cocultures of *G. metallireducens* and *G. sulfurreducens*, grown with extensive entangled structures formed by numerous conductive filamentous nanowires. With these filamentous nanowires, the syntrophic partners form tight aggregates that shorten the electron transfer distance between each other and reduce energy dissipation (Summers et al., 2010). In contrast, knockout of pilin expression genes in either *G. metallireducens* or *G. sulfurreducens* prevents tight aggregate formation and syntrophic electron exchange between them, which underscored the critical role of conductive nanowires in DIET. With the development of new characterization technologies, novel cytochrome-based conductive microbial nanowires as OmcS and OmcZ have been successively discovered in *G. sulfurreducens* (Shen et al., 2025; Wang et al., 2022, 2019; Yalcin et al., 2020). Mutating either of OmcS or OmcZ nanowire impaired the EET process and growth of *G. metallireducens* and *G. sulfurreducens* syntrophy, indicating that both OmcS and OmcZ play an important role in DIET (Jiang et al., 2023). DIET through conductive extracellular filaments is now considered a major mechanism in methanogenic aggregates, anaerobic digesters, rice paddy soils, and conductive mineral-rich environments (Figure 1A).

On the other hand, transmembrane proteins also play a role in DIET (Figure 1B). Phuc et al. discovered in a syntrophic anaerobic photosynthetic system involving *G. sulfurreducens* and *Prosthecochloris aestuarii* that the outer membranes of the two organisms are in close contact, and that *G. sulfurreducens* strains lacking the transmembrane porin-cytochrome complex (ombB-omaB-omcB-orfS-ombC-omaC-omcC) were unable to establish coculture with *P. aestuarii*, indicating that outer membrane proteins influence the DIET process between microorganisms (Ha et al., 2017).

In addition to relying on their own conductive structures for DIET, microorganisms can also achieve DIET with the assistance of exogenous abiotic conductive materials such as magnetite, granular activated carbon, or biochar (Figure 1C). Kato et al. (2012a) added (semi)conductive iron oxides, including magnetite and hematite, to rice paddy soil and found that electron transfer between *Geobacter* and *Methanosarcina* was enhanced, whereas the addition of non-conductive iron oxides did not promote electron transfer between them. Liu et al. (2015) reported that OmcS-deficient strain of *G. sulfurreducens*, which is unable to perform syntrophic electron exchange, could form cocultures with *G. metallireducens* upon the addition of exogenous magnetite, suggesting that magnetite can compensate for the loss of OmcS. Conductive carbon materials such as granular activated carbon, biochar, and carbon cloth can also mediate microbial DIET (van der Zee et al., 2003). Liu et al. (2012) demonstrated that, with the mediation of granular activated carbon, both the cytochrome OmcS-deficient strain and the pilin (PilA)-deficient strain of *G. sulfurreducens* could achieve syntrophic electron exchange with *G. metallireducens*, proving that granular activated carbon has the capacity to mediate DIET, and that DIET mediated by granular activated carbon does not require filamentous structures or extracellular cytochrome c.

Recently, a new way of DIET through cell fusion was discovered (Figure 1D). Liu et al. (2024) report that a Gram-positive nonelectroactive *Clostridium intestinale* attained EET-capability for ethanol metabolism only after forming a chimera with *G. sulfurreducens*. *C. intestinale* was not able to catabolize ethanol since it could not perform EET to discharge NADH, thus achieving intracellular redox balance. When grown with *G. sulfurreducens*, unable to catabolize ethanol as well, these two species formed an intimate connection via cell fusion by which the cytochrome dependent EET pathway of *G. sulfurreducens* was integrated with the oxidation of NADH in *C. intestinale*, providing *C. intestinale* an EET pathway for NADH oxidation and energy generation. Meanwhile, *G. sulfurreducens* transferred the electrons from *C. intestinale* to fumarate for energy production. This intimate cell fusion allows *C. intestinale* and *G. sulfurreducens* to perform DIET, which provides new perspectives for us to understand the energetic coupling between bacterial species and the ecology of microbial syntrophy.

### 2.2 Mediated interspecies electron transfer

Although *Geobacter* species are often discussed in terms of direct electron transfer, mediated routes also matter in anoxic soils (Figure 1). The MIET mechanism refers to the way in which microorganisms transfer electrons through the use of small molecule substances with redox activity that are naturally produced by themselves, synthetically produced, or artificially introduced (Figure 1E, Stams and Plugge, 2009). Rotaru et al. (2012) found that in a coculture system of *G. sulfurreducens* and *Pelobacter carbinolicus*, the growth of *P. carbinolicus* was inhibited when cocultured with Δ*hybL*Δ*fdnG*, formate dehydrogenase/hydrogenase double mutant of *G. sulfurreducens*, whereas its growth was not affected when cocultured with Δ*hybL*, hydrogenase deficient strain of *G. sulfurreducens*. This result indicated that the high expression of the formate dehydrogenase gene (*fdnG*) in *G. sulfurreducens* could compensate for the loss of hydrogenase in the coculture, thereby demonstrating that *P. carbinolicus* and *G. sulfurreducens* can simultaneously utilize both H_2_ and formate as IET carriers to achieve syntrophy.

In addition to H_2_ and formate, other redox active electron mediators can also mediate microbial MIET (Figure 1F). Huang et al. (2020) found that riboflavin-mediated MIET exists in a coculture of *G. metallireducens* and *G. sulfurreducens*. In this context, riboflavin could act as an electron shuttle to promote MIET between planktonic *Geobacter* species. Kaden et al. (2002) identified L-cystine/cysteine-mediated IET in a coculture of *G. sulfurreducens* and *Wolinella succinogenes*, with acetate as the electron donor and nitrate as the electron acceptor. In this syntrophy, *G. sulfurreducens* reduced L-cystine to L-cysteine while oxidizing acetate, and *W. succinogenes* utilized L-cysteine to reduce nitrate, during which L-cysteine was oxidized back to L-cysteine, which then participated again in acetate oxidation. In addition, Smith et al. (2015) found that the addition of the AQDS to syntrophy of *G. sulfurreducens* and *G. metallireducens* stimulated the syntrophic metabolic rate, and mutant coculture experiments demonstrated that AQDS-mediated IET could provide sufficient energy for the recipient microorganism *G. sulfurreducens* to support growth.

In soil microsites, *Geobacter*-based syntrophy may reduce humic substances or Fe-organic complexes, which then transfer electrons to minerals or partner organisms. Such processes extend electron flow beyond physical cell-cell contact and may be especially relevant in aggregated soils rich in organic matter and poorly crystalline Fe oxides.

## 3 *Geobacter*-based syntrophy in carbon cycling

**Figure 2:**
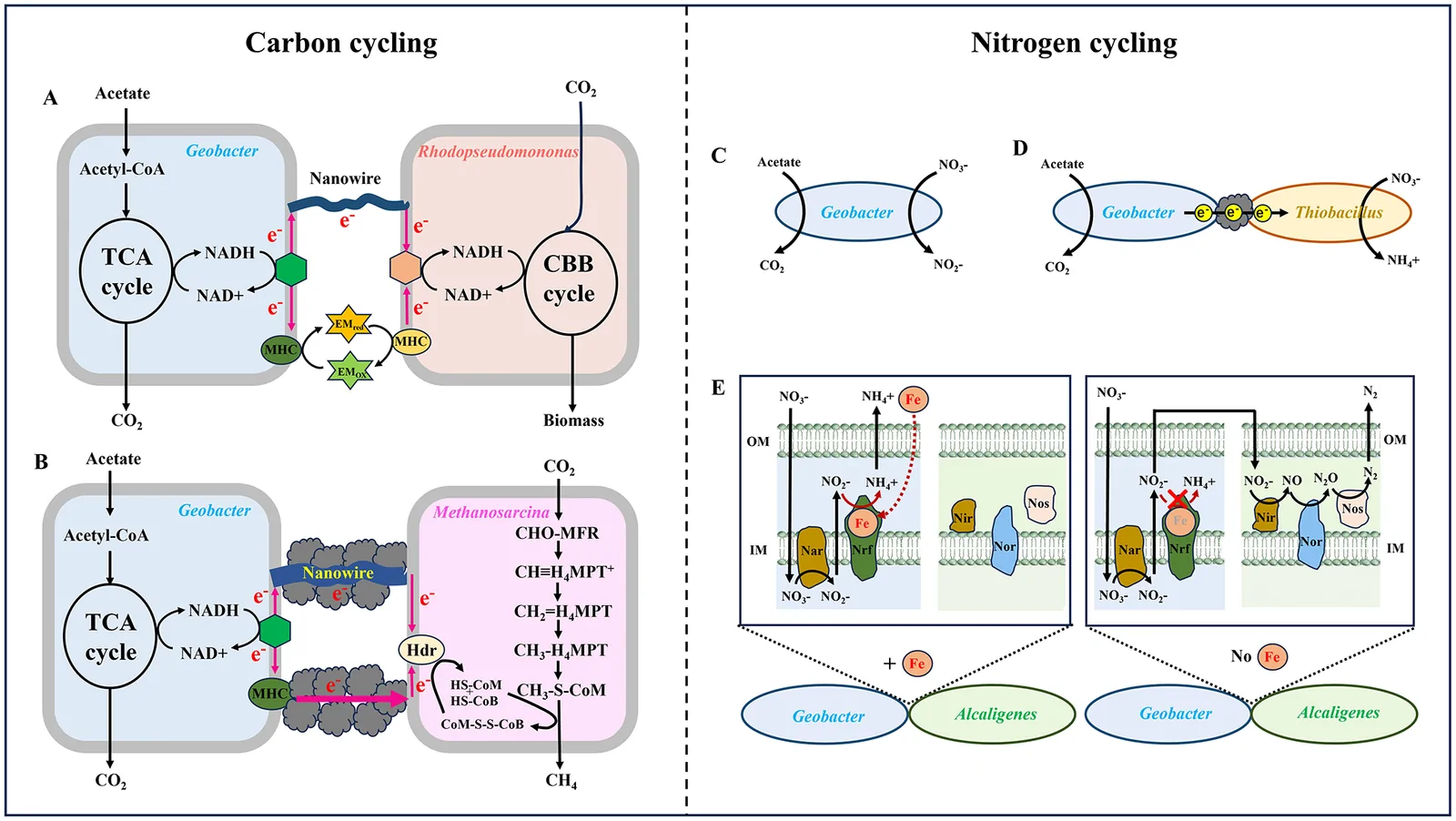
Carbon and nitrogen metabolism pathways of *Geobacter*-based syntrophy. **(A)** *Geobacter* and *Rhodopseudomonas* syntrophy. **(B)** *Geobacter* and *Methanosarcina* syntrophy. **(C)** *Geobacter* species directly reduce nitrate. **(D)** *Geobacter* species supply electrons to denitrifiers. **(E)** *Geobacter* species regulate nitrite reductase activity with Fe(III) to influence whether nitrate reduction ends in NH4^+^ or gaseous nitrogen (adapted from Zhan et al., 2025).

*Geobacte*r-based syntrophy influences the global carbon cycle by participating in multiple processes, including carbon decomposition and fixation metabolism, as well as methane metabolism (Butler et al., 2009). *Geobacter* species usually oxidizes acetate by the tricarboxylic acid (TCA) cycle (Butler et al., 2009). Recent studies have revealed that under dark and anoxic conditions, *G. metallireducens* can form syntrophy with *Rhodopseudomonas palustris* through nanowires or flavins, driving electroautotrophic growth of *R. palustris* (Figure 2A, Liu et al., 2021). In syntrophic anaerobic photosynthesis, electrons from acetate oxidation by *G. sulfurreducens* through TCA cycle were transferred to *P. aestuarii*, allowing the phototroph to use those electrons for CO_2_ fixation into biomass through the Calvin-Benson-Bassham (CBB) carbon fixation cycle. Cytochrome c2 is considered as the primary electron uptake protein in *R. palustris* to transport electrons inward through the periplasm. Furthermore, *Geobacter* transfers electrons directly to methanogens, including *Methanosaeta* and *Methanosarcina barkeri*, via DIET, thereby directly driving CO_2_ reduction to methane (Liu et al., 2025). Liu et al. found that the addition of capacitive materials strengthened the DIET of *Geobacter* and *Methanosarcina* (Figure 2B). In the syntrophy, *Geobacter* transferred electrons across outer membrane transmembrane protein complexes and then further to the attached capacitive material through extracellular c-type cytochromes and conductive nanowires. The capacitive materials facilitate electron transfer to *Methanosarcina* species, supporting carbon dioxide reduction and Hdr-related proteins in *Methanosarcina* species provide a pathway for the terminal electron transfer reaction in methanogenesis. Rotaru et al. (2014) demonstrated that in a coculture system with ethanol as the electron donor and CO_2_ as the electron acceptor, neither *G. metallireducens* nor *M. barkeri* could survive individually; however, when both were present together, simultaneous ethanol oxidation and CO_2_ reduction to methane occurred. Further investigation revealed that the two organisms rely on DIET to achieve CO_2_ fixation and methanogenesis. Holmes et al. (2021) revealed that *G. metallireducens* was able to form syntrophy with *Methanosarcina acetivorans* through DIET with multi-heme c-type cytochrome MmcA and presence of granular activated carbon could substitute the role of conductive nanowire in DIET. However, Zhou et al. (2023) found the addition of granular activated carbon amendments inhibited the growth of the syntrophy of *G. metallireducens* and *Methanothrix thermoacetophila*, but the addition of magnetite significantly enhanced the cell growth indicating the right choice of conductive material is important. Nonetheless, metagenomic and metatranscriptomic data analyses suggest that *Geobacter*-driven methanogenesis may be widespread in various methanogenic environments. The syntrophy between *Geobacter* and methanogens promotes methane emissions in anoxic environments, thereby influencing global warming.

Additionally, Yang et al. (2024) found that under ethanol-limited condition *G. metallireducens* and *G. sulfurreducens* tended to form syntrophy through DIET. To overcome electron donor limitation, c-type cytochromes in the syntrophy was actively released to extracellular environment, which indicated that protein movement in coordination with cellular needs. While Semenec et al. (2020) revealed that the syntrophic interaction between *G. sulfurreducens* and *Pseudomonas aeruginosa* would change over evolutionary time. As the syntrophy underwent serial transfers, *G. sulfurreducens* upregulated its formate dehydrogenase (FdnG) and hydrogenase (HybA) enzymes, allowing for formate or hydrogen to be utilized as electron donors and suggesting an increase in its metabolic flexibility. These findings provide new insights into the role of *Geobacte*r-based syntrophy in the biogeochemical cycling of carbon.

## 4 *Geobacter*-based syntrophy in nitrogen cycling

Nitrate reduction is usually a respiratory process of microorganisms in anoxic environments (Cole and Richardson, 2008). Nitrate can be reduced by *Geobacter*-based syntrophy to N_2_ through denitrification, causing nitrogen loss, or to NH4^+^ through dissimilatory nitrate reduction to ammonium (DNRA), conserving reactive nitrogen in soils (Wan et al., 2023). Denitrification through IET may play an important role in soil when the growing condition of *Geobacter* species and partner microorganisms was significantly changed. *G. sulfurreducens* was confirmed to be able to form syntrophy with *Diaphorobacter, Delftia*, and *Shinella* species to reduce nitrate to nitrogen gas (Wan et al., 2018). *G. sulfurreducens* has been also found to participate in the nitrogen cycle by establishing syntrophic relationships with *Thiobacillus denitrificans* to accomplish acetate oxidation coupled to dissimilatory nitrate reduction to ammonium with the presence of magnetite (Kato et al., 2012b). Additionally, in coculture of *G. metallireducens* with *Alcaligenes faecalis*, Zhan et al. (2025) found that the coculture performed DNRA under Fe(III)-replete conditions, while performing interspecies synergistic denitrification under Fe(III)-depleted conditions (Figure 2E). They identified that nitrate reduction partitioning in the coculture was governed by the nitrite reductase (Nrf) activity of *G. metallireducens*, which was Fe(III)-dependent. This provides a mechanistic link between Fe and N cycling. NrfA is a multiheme cytochrome c nitrite reductase that converts nitrite to ammonium. Because the catalytic function of Nrf depends on heme maturation and iron availability, ferruginous microsites may favor DNRA, whereas Fe limitation may force nitrite leakage to denitrifying partners. In this model, *Geobacter* forms substrate syntrophy with *A. faecalis* instead of electron syntrophy, and controls the enzymatic branch point between nitrogen retention and nitrogen loss. Under Fe(III)-replete conditions, the genes encoding nitrate reductase (Nar) and nitrite reductase (Nrf) were highly expressed in *G. metallireducens* conducting a nitrate ammonification pathway, in which condition *A. faecalis* hardly survived. Under Fe(III)-depleted condition, genes encoding nitrite reductase (Nir) and nitric oxide reductase (Nor) in *A. faecalis* were highly expressed. Otherwise, nitrite is diffused from *G. metallireducens* as diffusive intermediate and to *A. faecalis* for further reduction. Thereafter, a synergistic denitrification is established. Through DNRA, autogenous nitrogen fixation, and the stimulation of nitrogen metabolic activities in other microorganisms, *Geobacter* plays a considerable role in the biogeochemical cycling of nitrogen. Overall, *Geobacte*r-based syntrophy influences nitrogen cycling in at least three ways. First, *Geobacter* species directly reduce nitrate (Figure 2C) or participate in nitrate-reducing syntrophy. Second, *Geobacter* species supply electrons to synergistic partner (Figure 2D). Third, Fe cycling driven by *Geobacter* species regulate nitrite reductase activity and thereby influence whether nitrate reduction ends in NH4^+^ or gaseous nitrogen (Figure 2E).

## 5 *Geobacter*-based syntrophy in sulfur cycling

The sulfur cycle in anoxic soils is primarily driven by sulfate-reducing bacteria (SRB) that oxidize organic matter or H_2_ while reducing sulfate to sulfide (Jørgensen, 2021). SRB have synergistic effects in the sulfur cycle and form syntrophic associations, for instance, growing syntrophically with methanogens. Compared with carbon and nitrogen cycling, direct evidence for *Geobacter*-based syntrophy for sulfur cycling in soils is less developed. While the SRB in consortia are typically not *Geobacter*, the principle of DIET-mediated syntrophy in sulfur cycling extends to other partnerships. In enrichment cultures supplemented with both ferrihydrite and sulfate, a methanogenic archaeon (*Methanosarcina*) and iron- and sulfate-reducing bacteria (*Geobacter* and *Desulfotomaculum*, respectively) predominated (Kato and Igarashi, 2019). This suggests that *Geobacter* can coexist and potentially interact syntrophically with SRB in environments where both Fe(III) and sulfate are available as electron acceptors (Figure S1). The key research gap is whether *Geobacter* forms stable DIET partnerships with sulfate reducers, or sulfur oxidizers in soils. Current evidence supports strong indirect coupling through Fe-S mineral chemistry and selected laboratory syntrophies, but the molecular basis of *Geobacter*-based syntrophy in natural soils for sulfur cycling remains underdefined.

## 6 *Geobacter*-based syntrophy in iron cycling

Fe(III) oxides are widespread in soils and sediments, and microbial Fe reduction couples the oxidation of organic compounds to Fe(III) reduction (Bonneville et al., 2004). In complex anoxic soil communities, *Geobacter* often functions as part of syntrophic consortia that collectively drive Fe(III) reduction. It has been suggested that addition of biochar may stimulate microbial Fe(III) reduction and methanogenesis (Yuan et al., 2018). This stimulation was attributed to biochar functioning as both a “geobattery” (storing and releasing electrons) and a “geoconductor” (facilitating electron transfer between cells). The enrichment culture was dominated by Fe(III)-reducing *Geobacteraceae* and *Methanosarcina* taxa, suggesting the close coupling between iron reduction and methanogenesis in paddy soils. *Geobacter* directly reduces Fe(III) oxides using outer-membrane cytochromes, extracellular cytochromes, nanowires, and redox mediators. This process releases Fe(II), changes mineral crystallinity, alters surface reactivity, and affects the sorption or release of organic carbon.

In syntrophic communities, Fe(III) minerals can also act as shared electron acceptors or conductive conduits. For example, magnetite and other conductive minerals can facilitate electron transfer between *Geobacter* and methanogens or denitrifiers, effectively converting mineral surfaces into community-level electron buses (Dornelles et al., 2026). The Fe cycling also regulates N and S pathways. Fe(III) availability can favor DNRA by sustaining Nrf activity in *G. metallireducens*, whereas Fe limitation can shift nitrate reduction toward partner-driven denitrification. Fe(II) generated by *Geobacter* can react with sulfide, controlling sulfide toxicity, sulfur mineral formation. Thus, Fe is not simply an electron acceptor, but also a regulatory scaffold that determines electron allocation across C/N/S pathways.

## 7 Conclusion

*Geobacter* species, through their versatile IET machinery, encompassing both DIET and MIET routes, establish syntrophic consortia with partner microorganisms that are central to biogeochemical cycling in anoxic soils. These syntrophic interactions overcome thermodynamic constraints by coupling the oxidation of organic carbon to the reduction of terminal electron acceptors across microbial partners, thereby sustaining anaerobic carbon mineralization, methanogenesis, nitrate reduction, sulfate reduction, and Fe(III) reduction. Therefore, understanding *Geobacter*-based syntrophy is essential for predicting and managing C/N/S/Fe cycling in anoxic soils. We propose that *Geobacter*-based syntrophy should be viewed as a redox-partitioning mechanism, a dynamic control point that determines whether electrons derived from organic carbon are routed toward methane production, ammonium conservation, gaseous nitrogen loss, mineral reduction, or microbial biomass synthesis. This conceptual framework bridges microscale electron transfer events with macroscale elemental fluxes, offering a mechanistic understanding of how anoxic soil communities regulate multiple biogeochemical cycles simultaneously. Looking forward, major knowledge gaps remain: (i) the *in situ* prevalence and spatiotemporal dynamics of *Geobacter*-based syntrophy in natural soil aggregates; (ii) the regulatory network that governs electron allocation among competing C/N/S/Fe pathways under fluctuating redox conditions; and (iii) the potential to harness or engineer these syntrophic interactions for climate-smart agriculture (e.g., reducing methane emissions or enhancing nitrogen retention). Addressing these challenges will require integrated approaches combining high-resolution omics, stable isotope tracing, and biogeochemical modeling. Ultimately, a deeper understanding of *Geobacter*-based syntrophy will not only advance fundamental microbial ecology but also inform strategies for managing soil health and global biogeochemical cycles in a changing environment.
