# Supplementary material for: Geobacter-based syntrophy drives biogenic element cycling in anoxic soils: a review
Source: Front Microbiol. 2026 Sep 4;17:1932208. doi: 10.3389/fmicb.2026.1932208 (PMC13587344; doi:10.3389/fmicb.2026.1932208)
Supplement: Supplementary file 1 [file Supplementary_file_1.docx]

**Supporting information**

***Geobacter*****-based syntrophy drives biogenic element cycling in anoxic soils: A review**

Rong Tang^1^^†^, Huayuan Shangguan^2†^, Zhengli Yang^1^, Weiguo Zhang^3^*, Yin Ye^1^*

^1^ College of Ecology and Resources Engineering, Wuyi University, Wuyishan 354300, China

^2^ College of Tea and Food, Wuyi University, Wuyishan 354300, China

^3^ Engineering Research Center of Prevention and Control of Geological Disasters in the Mountainous Areas of Northern Fujian, Wuyi University, Wuyishan 354300, China

^†^These authors have contributed equally to this work

***Correspondence:**

Yin Ye, E-mail: [yinye@wuyiu.edu.cn](mailto:yinye@wuyiu.edu.cn)

Weiguo Zhang, E-mail: [zhangweiguo@wuyiu.edu.cn](mailto:zhangweiguo@wuyiu.edu.cn)

**Table S1**

Summary of *Geobacter*-based syntrophy mechanisms.

| Electron donor microorganism | Electron acceptor microorganism | DIET/MIET | Mediators | Reference |
| --- | --- | --- | --- | --- |
| *G. metallireducens* | *G. sulfurreducens* | DIET/MIET | Conductive nanowire, cytochrome, biochar, carbon cloth, magnetite; riboflavin, AQDS | (Chen et al., 2014a; Chen et al., 2014b; Huang et al., 2020; Liu et al., 2015; Smith et al., 2023; Summers et al., 2010) |
| *G. metallireducens* | *Methanosarcina barkeri* | DIET | Conductive nanowire, cytochrome, biochar, carbon cloth, magnetite | (Chen et al., 2014b; Holmes et al., 2022; Rotaru et al., 2014b; Wang et al., 2018) |
| *G. metallireducens* | *Methanosarcina acetivorans* | DIET | Cytochrome, granular activated carbon | (Holmes et al., 2022; Holmes et al., 2021) |
| *G. metallireducens* | *Methanotrix harundinacea* | DIET | Conductive nanowire | (Rotaru et al., 2014a) |
| *G. metallireducens* | *Methanobacterium sp. YSL* | DIET | Granular activated carbon, carbon cloth | (Zheng et al., 2020) |
| *G. metallireducens* | *Methanotrix thermosacetophila* | DIET | Cytochrome, magnetite | (Zhou et al., 2023) |
| *G. metallireducens* | *R. palustris* | DIET | Conductive nanowire, cytochrome | (Liu et al., 2021) |
| *C. intestinale* | *G. sulfurreducens* | DIET | Cytochrome | (Liu et al., 2024) |
| *G. sulfurreducens* | *P. aestuarii* | DIET | Conductive nanowire | (Ha et al., 2017) |
| *G. sulfurreducens* | *T. denitrificans* | DIET | Conductive mineral | (Kato et al., 2012) |
| *G. sulfurreducens* | *W. succinogenes* | MIET | L-cystine/cysteine | (Kaden et al., 2002) |
| *G. sulfurreducens* | *P. carbinolicus* | MIET | H_2_, formate | (Rotaru et al., 2012) |
| *G. metallireducens* | *W. succinogenes* | MIET | AQDS | (Lovley et al., 1999) |

Figure S1 Sulfur and iron metabolism pathways of *Geobacter*-based syntrophy.

**Reference**

Chen S., Rotaru A.E., Liu F., Philips J., Woodard T.L., Nevin K.P., et al. (2014a). Carbon cloth stimulates direct interspecies electron transfer in syntrophic co-cultures. *Bioresour Technol*. 173, 82-86. doi: 10.1016/j.biortech.2014.09.009

Chen S., Rotaru A.E., Shrestha P.M., Malvankar N.S., Liu F., Fan W., et al. (2014b). Promoting interspecies electron transfer with biochar. *Sci. Rep.* 4, 5019. doi: 10.1038/srep05019

Ha P.T., Lindemann S.R., Shi L., Dohnalkova A.C., Fredrickson J.K., Madigan M.T., et al. (2017). Syntrophic anaerobic photosynthesis via direct interspecies electron transfer. *Nat Commun*. 8, 13924. doi: 10.1038/ncomms13924

Holmes D.E., Zhou J., Smith J.A., Wang C., Liu X., Lovley D.R. (2022). Different outer membrane c-type cytochromes are involved in direct interspecies electron transfer to *Geobacter* or *Methanosarcina* species. *mLife*. 1(3), 272-286. doi: 10.1002/mlf2.12037

Holmes D.E., Zhou J., Ueki T., Woodard T., Lovley D.R.J.M. (2021). Mechanisms for electron uptake by *Methanosarcina* acetivorans during direct interspecies electron transfer. *mBio*. 12(5), 10.1128/mbio. 02344-21. doi: 10.1128/mbio.02344-21

Huang L., Liu X., Ye Y., Chen M., Zhou S. (2020). Evidence for the coexistence of direct and riboflavin-mediated interspecies electron transfer in *Geobacter* co-culture. *Environ Microbiol*. 22(1), 243-254. doi: 10.1111/1462-2920.14842

Kaden J., A S.G., Schink B. (2002). Cysteine-mediated electron transfer in syntrophic acetate oxidation by cocultures of *Geobacter sulfurreducens* and *Wolinella succinogenes*. *Arch Microbiol*. 178(1), 53-8. doi: 10.1007/s00203-002-0425-3

Kato S., Hashimoto K., Watanabe K. (2012). Microbial interspecies electron transfer via electric currents through conductive minerals. *Proceedings of the National Academy of Sciences*. 109(25), 10042-10046. doi: 10.1073/pnas.1117592109

Liu F., Rotaru A.E., Shrestha P.M., Malvankar N.S., Nevin K.P., Lovley D.R. (2015). Magnetite compensates for the lack of a pilin-associated c-type cytochrome in extracellular electron exchange. *Environ Microbiol*. 17(3), 648-55. doi: 10.1111/1462-2920.12485

Liu X., Huang L., Rensing C., Ye J., Nealson K.H., Zhou S. (2021). Syntrophic interspecies electron transfer drives carbon fixation and growth by *Rhodopseudomonas palustris* under dark, anoxic conditions. *Sci Adv*. 7(27), eabh1852. doi: 10.1126/sciadv.abh1852

Liu X., Ye Y., Yang N., Cheng C., Rensing C., Jin C., et al. (2024). Nonelectroactive *Clostridium* obtains extracellular electron transfer-capability after forming chimera with *Geobacter*. *ISME Commun*. 4(1), ycae058. doi: 10.1093/ismeco/ycae058

Lovley D.R., Fraga J.L., Coates J.D., Blunt‐Harris E.L.J.E.m. (1999). Humics as an electron donor for anaerobic respiration. *Environ Microbiol*. 1(1), 89-98. doi: 10.1046/j.1462-2920.1999.00009

Rotaru A.-E., Shrestha P.M., Liu F., Shrestha M., Shrestha D., Embree M., et al. (2014a). A new model for electron flow during anaerobic digestion: Direct interspecies electron transfer to *Methanosaeta* for the reduction of carbon dioxide to methane. *Energy Environ Sci*. 7(1), 408-415. doi: 10.1039/c3ee42189a

Rotaru A.E., Shrestha P.M., Liu F., Markovaite B., Chen S., Nevin K.P., et al. (2014b). Direct interspecies electron transfer between *Geobacter metallireducens* and *Methanosarcina barkeri*. *Appl Environ Microbiol*. 80(15), 4599-605. doi: 10.1128/AEM.00895-14

Rotaru A.E., Shrestha P.M., Liu F., Ueki T., Nevin K., Summers Z.M., et al. (2012). Interspecies electron transfer via hydrogen and formate rather than direct electrical connections in cocultures of *Pelobacter carbinolicus* and *Geobacter sulfurreducens*. *Appl Environ Microbiol*. 78(21), 7645-51. doi: 10.1128/aem.01946-12

Smith J.A., Holmes D.E., Woodard T.L., Li Y., Liu X., Wang L.-Y., et al. (2023). Detrimental impact of the *Geobacter metallireducens* type VI secretion system on direct interspecies electron transfer. *Microbiology Spectrum*. 11(5), e00941-23. doi: 10.1128/spectrum.00941-23

Summers Z.M., Fogarty H.E., Leang C., Franks A.E., Malvankar N.S., Lovley D.R. (2010). Direct exchange of electrons within aggregates of an evolved syntrophic coculture of anaerobic bacteria. *Sci*. 330(6009), 1413-5. doi: 10.1126/science.1196526

Wang O., Zheng S., Wang B., Wang W., Liu F. (2018). Necessity of electrically conductive pili for methanogenesis with magnetite stimulation. *PeerJ*. 6, e4541. doi: 10.7717/peerj.4541

Zheng S., Liu F., Wang B., Zhang Y., Lovley D.R. (2020). *Methanobacterium* capable of direct interspecies electron transfer. *Environ Sci Technol*. 54(23), 15347-15354. doi: 10.1021/acs.est.0c05525

Zhou J., Smith J.A., Li M., Holmes D.E.J.M. (2023). Methane production by *Methanothrix thermoacetophila* via direct interspecies electron transfer with *Geobacter metallireducens*. *Environ Microbiol*. 14(4), e00360-23. doi: 10.1128/mbio.00360-23
